# Cellular senescence in hepatocytes contributes to metabolic disturbances in NASH

**DOI:** 10.3389/fendo.2022.957616

**Published:** 2022-08-22

**Authors:** Laurianne Bonnet, Ida Alexandersson, Ritesh K. Baboota, Tobias Kroon, Jan Oscarsson, Ulf Smith, Jeremie Boucher

**Affiliations:** ^1^ Wallenberg Centre for Molecular and Translational Medicine, University of Gothenburg, Gothenburg, Sweden; ^2^ The Lundberg Laboratory for Diabetes Research, Department of Molecular and Clinical Medicine, Sahlgrenska Academy, University of Gothenburg, Gothenburg, Sweden; ^3^ Bioscience Metabolism, Research and Early Development, Cardiovascular, Renal and Metabolism (CVRM), BioPharmaceuticals R&D, AstraZeneca, Gothenburg, Sweden; ^4^ Late Stage Development, Cardiovascular, Renal and Metabolism (CVRM), BioPharmaceuticals R&D, AstraZeneca, Gothenburg, Sweden

**Keywords:** senescence, NAFLD, NASH, hepatocytes, insulin signaling, metabolism

## Abstract

Cellular senescence is a state of irreversible cell cycle arrest and has been shown to play a key role in many diseases, including metabolic diseases. To investigate the potential contribution of hepatocyte cellular senescence to the metabolic derangements associated with non-alcoholic steatohepatitis (NASH), we treated human hepatocyte cell lines HepG2 and IHH with the senescence-inducing drugs nutlin-3a, doxorubicin and etoposide. The senescence-associated markers p16, p21, p53 and beta galactosidase were induced upon drug treatment, and this was associated with increased lipid storage, increased expression of lipid transporters and the development of hepatic steatosis. Drug-induced senescence also led to increased glycogen content, and increased VLDL secretion from hepatocytes. Senescence was also associated with an increase in glucose and fatty acid oxidation capacity, while *de novo* lipogenesis was decreased. Surprisingly, cellular senescence caused an overall increase in insulin signaling in hepatocytes, with increased insulin-stimulated phosphorylation of IR, Akt, and MAPK. Together, these data indicate that hepatic senescence plays a causal role in the development of NASH pathogenesis, by modulating glucose and lipid metabolism, favoring steatosis. Our findings contribute to a better understanding of the mechanisms linking cellular senescence and fatty liver disease and support the development of new therapies targeting senescent cells for the treatment of NASH.

## Introduction

Non-alcoholic fatty liver disease (NAFLD) is a complex and multifactorial disease affecting approximately 25% of the general population (1). It ranges from non-alcoholic fatty liver (NAFL), characterized by a buildup of lipids in hepatocytes, to the more advanced non-alcoholic steatohepatitis (NASH) with inflammation, hepatocyte ballooning, and different degrees of fibrosis (2). Both NAFL and NASH are commonly associated with obesity and type 2 diabetes (1, 3, 4), while age is also a major risk factor in the development of NASH (5).

Cellular senescence is defined by permanent and irreversible cell cycle arrest, and is a hallmark of ageing (6). Senescence can be induced by mitochondrial dysfunction, telomere shortening, and genotoxic and oxidative stress (7). Senescent cells are characterized by morphological alterations, disruption in nuclear membrane due to the loss of the nuclear lamina protein Lamin B1, increased activity of senescence associated β-galactosidase, and increased expression of cell cycle inhibitors such as p16^INK4A^, p21^Cip1^ and p53 (8). Another feature of senescent cells is the development of a characteristic secretome which includes inflammatory cytokines, growth factors and proteases, commonly referred to as the senescence-associated secretory phenotype (SASP). The SASP is pro-inflammatory and can contribute to senescence-induced tissue dysfunction in a paracrine manner (9).

NAFLD has recently been associated with increased senescent cell burden in the liver, both in rodents and humans (10–13). In healthy individuals, around 3–7% of hepatocytes are senescent but in end-stage liver disease the percentage can increase to 50–100% (11). Senescence in human hepatocytes causes a change in the secretory profile which promotes macrophage migration (14), and senescence has been shown to cause mitochondrial dysfunction and increase liver fat in mice (12). We have recently shown that hepatic senescence is associated with clinical progression of NAFLD/NASH, and correlates with disease severity (Baboota et al. Nature Metabolism, in press).

In the current study, we investigated the potential contribution of hepatocyte cellular senescence in the development of NASH. We found that treatment of human hepatocytes with senescence-inducing drugs caused profound changes in hepatocyte metabolism, with increased lipid and glycogen storage, enhanced insulin signaling, and increased VLDL secretion.

## Materials and methods

### Cell culture conditions and treatments

Human hepatocellular carcinoma HepG2 cells (ATCC, Manassas, VA, USA) and immortalized human hepatocyte IHH cells (provided by Prof Jan Boren at University of Gothenburg) were cultured in DMEM high glucose (Gibco, 41965039) and William’s E Medium GlutaMAX (Gibco, 32551087), respectively, and supplemented with 10% FBS (ThermoFisher, 10270-106) and 1% penicillin/streptomycin (ThermoFisher 15140-122). Medium was changed every 3 days. The cells were grown in cell culture incubators at 37°C in a humid environment, with 5% CO_2_.

To induce senescence, cells were treated with either nutlin-3a (Sigma-Merck, SML0580), etoposide (Sigma-Merck, E1383) or doxorubicin (Merck Millipore, 324380). For the 5 day treatment experiments, medium was changed after 3 days. Control cells were treated with 0.03% DMSO (Sigma-Merck, D8418). In some experiments, in order to create pro-steatotic conditions, 200 µM of oleic acid (Sigma-Merck, O1257) for IHH or 400 µM for HepG2 was added to culture medium containing 1% FBS (for both the –OA and +OA conditions) for 24h, after the treatment with the senescence inducing drugs.

For acute insulin/IGF1 signaling experiments, cells were incubated in 1% FBS medium for 4h, and then treated with 10 nM of human recombinant insulin (Actrapid Penfill; Novo Nordisk, Bagsvard, Denmark) or IGF1 (Thermo Fisher, PHG0078) for 10 min. Cells were then washed once with PBS and lysed in lysis buffer for Western blot analysis. For chronic insulin treatment experiments, IHH cells were treated for 5 days in regular culture medium with 10% FBS, supplemented with 100 nM of human recombinant insulin.

### Quantitative real-time PCR

Total RNA was isolated using the E.Z.N.A. Total RNA Kit (Omega Bio-tek, R6834-02), following the manufacturer’s instructions. cDNA was synthesized using the High Capacity cDNA Reverse Transcription Kit (Thermo Fisher Scientific, 4368813). PCR was performed using a TRIO Thermoblock Heat Cycler (Biotron Biometra). Gene expression was measured using a QuantStudio 6 Flex (Applied Biosystems, Foster City, CA) using either SYBR Green Master Mix (ThermoFisher, A25741) or TaqMan Fast Advanced Master Mix (Thermo Fisher Scientific, 4444557). The primer sequences used are listed in **Supplemental Table 1**, except for *CDKN2A* which was measured using Assay On-Demand (Thermo Fisher Scientific, Hs00923894_m1).

### Immunoblotting

Cells were lyzed in a buffer containing 10 mM NaF, 0.25 M EGTA, 25 mM NaCl, 1% IGEPAL, 1 mM sodium ortho-vanadate (Sigma-Merck, S6508), 25 mM Tris-HCl, in the presence of protease inhibitors (Sigma-Merck, S8820). 2,4 mU/µL benzonase nuclease (Merck, E1014-5KU) was added for 15 min at room temperature (RT) to reduce sample viscosity. Samples were then centrifuged at 12 000 rpm for 15 min at 4°C to sediment the insoluble material. The lysate was transferred to a new tube and frozen at -80°C for storage. The protein content was determined using Pierce Micro BCA protein assay (Thermo Fisher Scientific, 23227). Protein samples were prepared with 4X LDS Sample Buffer (ThermoFisher, NP00007) and 10X Sample Reducing Agent (ThermoFisher, NP00009), and denatured by heating at 95°C for 5 min. 20-25 µg of proteins were separated by electrophoresis on 4–12% Bis-Tris NuPAGE gels (Thermo Fisher, NP0336BOX) in 1X of MOPS buffer (ThermoFisher, NP0001) for 1 h at 120-140 V. The proteins were transferred to PVDF membranes previously activated in methanol for 1 min (BioRad, 1620177), in a transfer buffer (Tris Glycine buffer (10X, BioRad, 1610771), 10% ethanol) at 100 V for 1 to 2 h. Membranes were blocked for 30 min with StartingBlock™ (PBS) Blocking Buffer (Thermo Fisher, 37538) and incubated with primary antibodies overnight at 4°C. The membranes were then washed 3x 5 min in PBS-Tween and incubated with the secondary antibody for 1h at RT. The bands were visualized using ECL reagents (BioRad, 1705060 or 1705062). The list of primary and secondary antibodies used in this study is presented in **Supplemental Table 2**. Image lab software were used to quantify the bands.

### Immunofluorescence assay

Cells were cultured on coverslips for 5 days in 12-well plates, then fixed with 4% paraformaldehyde for 20 min at RT, followed by washing in PBS and permeabilization using 0.1% Triton X-100 in PBS for 5 min. After washing with PBS, cells were blocked for 10 min in blocking buffer (5% BSA/PBS) at RT. Cells were subsequently incubated overnight at 4°C with primary antibody against p21 (**Supplemental Table 2**, 1:300) in 1% BSA/PBS. After washing with PBS, cells were incubated with Alexa488-probed secondary antibody (Invitrogen, A-11008) for 1h followed by incubation with DAPI (Sigma-Merck, D9542, 1:5000) for 10 min. Each coverslip was inverted onto a slide containing 1 drop of mounting/antifade medium (Invitrogen, P36970) and stored at 4°C in the dark until viewing. Images were acquired using the Axio Observer 7 (Zeiss). p21 staining intensity was analyzed with the ImageJ software (NIH). Cells were then stained with DAPI and difference in cell number were corrected using DAPI fluorescence (measured at excitation 350 nm and at emission 450/490 nm) to normalize the p21 signal intensity in each coverslip. Images were taken using a bright field microscope (Zeiss Axio Vert). The percentage of cells with low, medium, and high p21 intensity was determined visually in a blinded manner.

### Oil red O staining

Cells were fixed in 4% paraformaldehyde (PFA) for 30 min. After washing with water, cells were incubated with 0.5% (wt/v) Oil Red O (Sigma-Aldrich, O1391) in 60% (v/v) isopropanol for 45 min at RT. Cells were washed with water and pictures were acquired using the Eclipse TS100 microscope (Nikon). Oil Red O quantification was measured by lysing the cells in 100% isopropanol for 10 min under shaking, and the absorbance of the Oil Red O dye was measured at 492 nm in a spectrophotometer (SpectraMax i3x, Molecular Devices). Cells were also stained with DAPI and differences in cell number were corrected using DAPI fluorescence (measured at excitation 350 nm and at emission 450/490 nm) to normalize the ORO intensity per well. Images were taken using a bright field microscope (Zeiss Axio Vert).

### Triglyceride content

Cells were scraped and collected in PBS (200 µL per well in a 6-well plate format), and were centrifuged at 12500 rpm at 4°C for 10 min and the buffer was discarded by pipetting. Triglycerides were extracted by incubating samples with 200 µL of cold 100% isopropanol at 4°C for 2 h. After a centrifugation for 5 min at 1300 x g at 4°C, 250 µL of supernatant was used to analyze triglycerides on ABX Pentra 400 according to the method for triglyceride quantification in the liver. Another plate of cells (cultured in same conditions) were stained with DAPI and differences in cell number were corrected using DAPI fluorescence (measured at excitation 350 nm and at emission 450/490 nm). Images were taken using a bright field microscope (Zeiss Axio Vert).

### VLDL secretion

After 5 days of treatment with senescence-inducing drugs, IHH cells were washed with PBS and incubated for 24h in low serum media (William’s E Medium supplemented with 1% penicillin/streptomycin, 1% of FBS and 1% of BSA) with oleic acid (200 µM). After the 24h OA treatment, cell culture media was collected and TG and ApoB100 levels in the media were measured using Triglyceride quantification colorimetric/fluorometric kit (Merck/Sigma, MAK266-1KT) and Human apoB kit (Mabtech, 3715-1HP-2), according to the manufacturer’s instructions. TG and ApoB100 quantification was normalized to total protein levels per well.

### Glucose production

After 5 days of treatment with senescence-inducing drugs, hepatocytes were washed in PBS and incubated in low serum William’s E medium containing 1% FBS and 0,5% of BSA for 4h. Cells were subsequently washed in PBS and cultured in Williams’ Medium E, w/L-Glutamine w/o Glucose (SMS gruppen, W1105-05) containing 1% FBS and 0,5% of BSA for 30 min, 2h and 6h. Cell culture media was collected and the concentration of glucose in the diluted media (1:2) was measured using the HK glucose assay (Sigma, GAHK-20) according to the manufacturer’s instructions and were normalized to total protein levels per well.

### Glycogen content

Senescent cells were incubated in low glucose medium (5 mM of glucose, made with 54,6% of Williams’ Medium E, w/L-Glutamine w/o Glucose (SMS gruppen, W1105-05) and 44,4% of William’s E Medium, GlutaMAX (SMS gruppen, W1105-05) each containing 1% of penicillin/streptomycin), supplemented with 1% BSA and in presence of 1 mM of carnitine for 4h. Glycogen was extracted according to Nagarajan et al. (15). Briefly, cells were scraped and lysed in 1 M KOH and heated at 65°C for 30 min. Lysates were then diluted in saturated Na_2_SO_4_ and absolute ethanol followed by centrifugation at 15,000 g for 15 min at 4°C. The supernatant was discarded, and the pellet was resuspended in 200 µL of water then adding 1.8 mL of ethanol (95%). The supernatant was discarded once more after a second centrifugation step (15,000 g for 15 min at 4°C) and the glycogen pellet was incubated with amyloglucosidase (Sigma, A-1602) overnight at 37°C in 1 ml of 0.25 M acetate buffer. Samples were vortexed and glycogen was measured with a HK glucose assay (Merck, GAHK20-1KT). Results were normalized to total protein levels per well.

### Lipid tracer studies

Cells were preincubated with oleic acid treatment (200 µM) for 16h in low serum medium (1% of FBS), and then treated for 4h with 0,5 µCi/mL of ^14^C oleic acid (Perkin Elmer, NEC317050UC) and 200 µM of cold oleic acid in low glucose medium (5 mM of glucose, made with 54,6% of Williams’ Medium E, w/L-Glutamine w/o Glucose (SMS gruppen, W1105-05) and 44,4% of William’s E Medium, GlutaMAX (SMS gruppen, W1105-05) each containing 1% of penicillin/streptomycin), supplemented with 1% BSA and 1 mM of carnitine (Calbiochem, #217525). Then, the specific protocols below were followed:

To measure oleate storage in intracellular lipids, cells were washed and collected in cold PBS in glasses tubes. A Folch extraction of cellular lipids was done according to the protocol established by Folch et al. (16). In short, after a centrifugation at 13 000 rpm at 4°C for 15 min, the cell pellet was lyzed in 1 mL of Chloroform/MetOH (2:1; v/v). The separation of phases was obtained after addition of water and centrifugation at 1000 g for 10 min at 4°C. The ^14^C oleate incorporation into lipids was measured by quantification of the radioactivity in the organic phase (lower phase) with a Tri-Carb 2800TR β-counter (PerkinElmer). Results were normalized to total protein levels per well.

To measure oleate oxidation, cell culture media was collected in a 15 mL tube and frozen at -20°C immediately for further analysis. An equal volume of 1 M perchloric acid was then added to the culture media and incubated overnight at 37°C under agitation. The released ^14^CO_2_ was trapped in a 1 N sodium hydroxide Whatman paper (Merck, WHA3030917) placed on a top of the tube (acting as a filter in a closed system), according to the protocol by Huynh et al. (17). The radioactivity from the filter was quantified with a Tri-Carb 2800TR β-counter. Results were normalized to total protein levels per well.

Oleate uptake was calculated as the sum of the amount of oleate stored into intracellular lipids and of oleate oxidation.

### Glucose tracer studies

Cells were incubated with 0,2 µCi/mL of D-[^14^C(U)]-Glucose (Perkin Elmer, NECO042x050UC) in low glucose medium (5 mM of glucose, made with 54,6% of Williams’ Medium E, w/L-Glutamine w/o Glucose (SMS gruppen, W1105-05) and 44,4% of William’s E Medium, GlutaMAX (SMS gruppen, W1105-05) each containing 1% of penicillin/streptomycin), in low serum medium (1% of FBS), supplemented with 1% BSA and in presence of 1 mM of carnitine for 4h.

To measure glucose oxidation, culture media was collected in a 15 mL tube and frozen at -20°C immediately for further analysis. The frozen media was then incubated with an equal volume of 1 M perchloric acid overnight at 37°C under agitation, according to the protocol by Huynh et al. (17). After a centrifugation at maximum speed for 10 min, the radioactivity was quantified in upper phase of the acid solution with a Tri-Carb 2800TR β-counter. Results were normalized to total protein levels per well.

To quantify *de novo* lipogenesis, cells were incubated with 0,2 µCi/mL of D-[^14^C(U)]-Glucose (Perkin Elmer, NECO042x050UC) in low glucose medium (5 mM of glucose, made with 54,6% of Williams’ Medium E, w/L-Glutamine w/o Glucose (SMS gruppen, W1105-05) and 44,4% of William’s E Medium, GlutaMAX (SMS gruppen, W1105-05) each containing 1% of penicillin/streptomycin), in low serum medium (1% of FBS) supplemented with 1% BSA, 1 mM of carnitine and 100 nM of insulin for 4h. Glucose incorporation into lipids was measured by quantification of the radioactivity in the organic phase after Folch extraction as described above and according to the protocol by Folch et al. (16) with a Tri-Carb 2800TR β-counter. Results were normalized to total protein levels per well

### Statistical analyses

The statistical analyses were performed using Prism 9 (GraphPad). Student’s two-tailed t test was performed for paired comparisons, and one-way ANOVA with Tukey’s test for multiple value comparisons of several groups. All data is reported as mean ± SEM several independent experiments, each performed with several replicates. A p-value less than 0.05 was considered significant.

## Results

### Induction of cellular senescence in human hepatocytes

**Figure 1:**
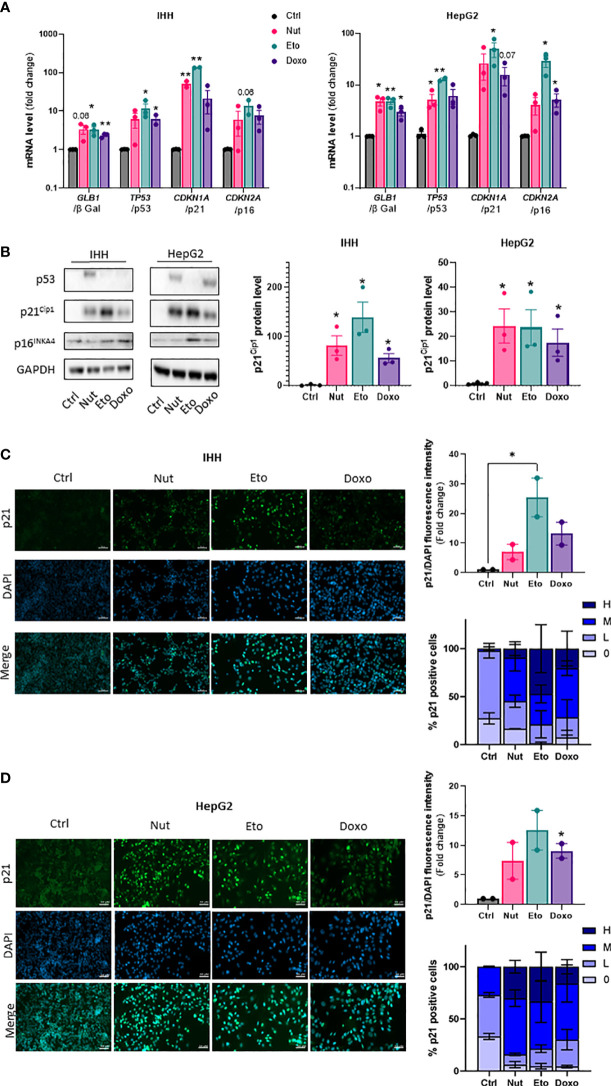
Nutlin-3a, etoposide and doxorubicin treatment increase senescence markers in human hepatocytes. IHH and HepG2 cells were treated with nutlin-3a (Nut; 3 µM), etoposide (Eto; 20 µM) and doxorubicin (Doxo; 0,2 µM) for 5 days. **(A)** mRNA expression of senescence markers normalized to *GAPDH*. **(B)** Western blot analysis of senescence markers p53, p21^Cip1^ and p16^INK4a^, and GAPDH as a loading control. Quantification of p21 protein levels were normalized GAPDH levels. **(C)** p21 immunofluorescence in IHH and **(D)** HepG2 cells. Hepatocytes were stained for p21 (green) and nuclei (DAPI, blue). Scale bars represents 50 µm. p21 fluorescence intensity was quantified with Image J and normalized to DAPI staining. The percentage of p21 positive cells was determined based on the degree of p21 fluorescence intensity from an average of 6 different images per group (H = high, M = medium, L = low, 0 = no p21 fluorescent signal detected). Results are expressed as Mean ± SEM. *P < 0.05, **P < 0.01 indicates a significant difference compared to control cells.

To induce cellular senescence in human hepatocytes, human hepatocellular carcinoma HepG2 cells and immortalized human hepatocytes (IHH cells) were treated with several compounds which have been shown to induce senescence *in vitro*: nutlin-3a (Nut), a small-molecule inhibitor of MDM2 (18) and etoposide (Eto) and doxorubicin (Doxo), two DNA-damaging agents (19, 20). Pilot experiments with different drug doses and incubation times were tested to find optimal conditions that would robustly induce senescence without causing toxicity in both HepG2 and IHH cells. Cellular senescence is associated with an increase in expression of p53, p21 and p16 and β galactosidase (8, 21, 22). Treatment of human hepatocyte HepG2 and IHH cells with 3 µM Nut, 20 µM Eto and 0,2 µM Doxo for 5 days caused modifications in cell shape with a flattened and elongated morphology characteristic of cellular senescence, and induced a robust increase in p53, p21 and p16, both at the mRNA (**Figure 1A**) and at the protein level (**Figure 1B**). Etoposide induced a 130-fold and 75-fold increase in *CDKN1A* mRNA levels (encoding the p21 protein) in IHH and HepG2 cells, respectively, while p21 protein levels were increased by 170-fold and 25-fold. Similar results were observed when cells were treated with Nut, Eto, and Doxo for 3 days (**Supplementary Figure 1**). The increase in p21 protein levels by the senescence-inducing drugs was also shown by immunocytochemistry. Detailed analysis further revealed that Nut, Eto and Doxo treatment caused both an increase in the number of p21 positive cells as well as an increase in the intensity of p21 staining. The percentage of cells with medium or high p21 staining went from 2% in control cells to 55-80% in senescent IHH cells, and from 27% in control cells to 70-84% in senescent HepG2 cells (**Figures 1C, D**). These results show that senescence was observed in a majority of cells.

### Cellular senescence increases steatosis in human hepatocytes

**Figure 2:**
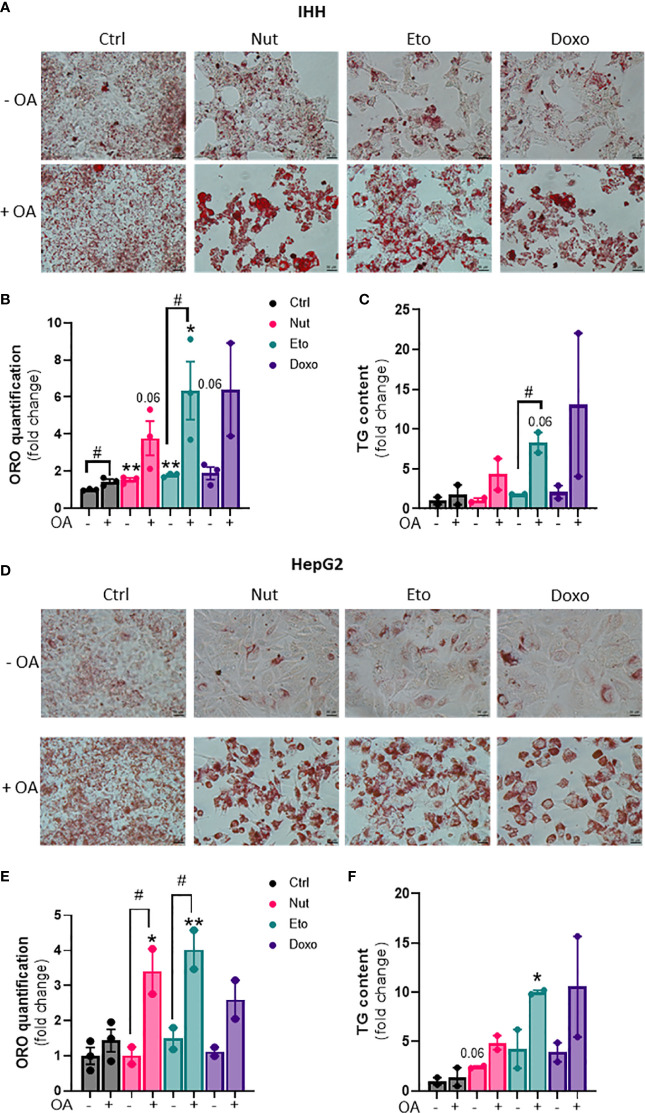
Increased steatosis in senescent human hepatocytes. Human hepatocytes were treated with nutlin (Nut; 3 µM), etoposide (Eto; 20 µM) and doxorubicin (Doxo; 0,2 µM) for 5 days, with or without the addition of oleic acid (OA) for another 24h. Oil Red O (ORO) staining **(A, D)**, ORO quantification **(B, E)** and triglyceride (TG) content **(C, F)** in IHH and HepG2 hepatocytes. Scale bars represent 25 µm. ORO quantification and TG levels were normalized to cell number with DAPI staining. Results are expressed as Mean ± SEM. *P < 0.05, **P < 0.01 indicates a significant difference compared to control cells. ^#^P < 0.05 indicates a significant difference compared to similar treatment condition without OA.

**Figure 3:**
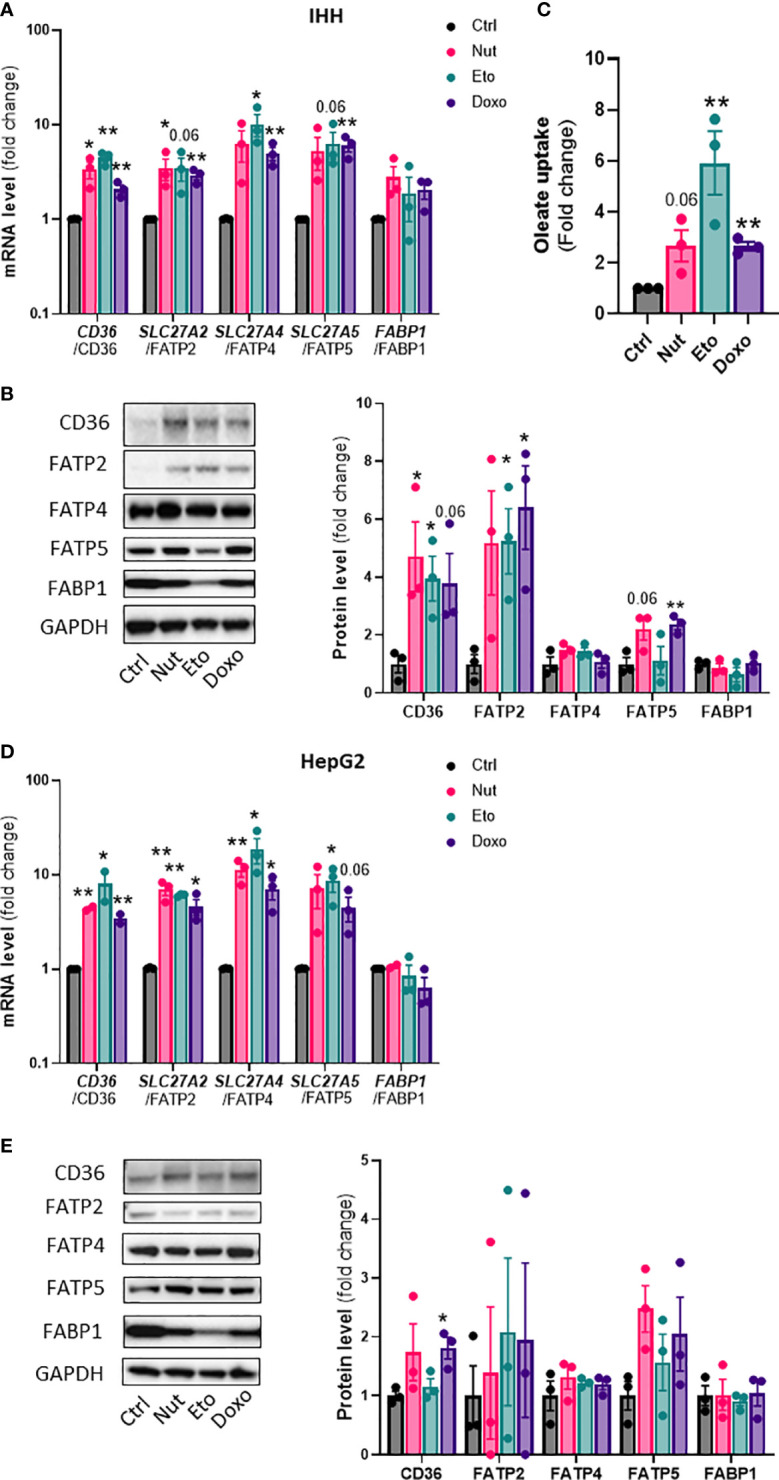
Increased expression of lipid transporters and fatty acid uptake in senescent human hepatocytes. Human hepatocytes were treated with nutlin (Nut; 3 µM), etoposide (Eto; 20 µM) and doxorubicin (Doxo; 0,2 µM) for 5 days. mRNA expression of lipid transporters normalized to GAPDH in IHH **(A)** and HepG2 **(D)** cells. Western blot analysis and quantification of lipid transporter protein levels normalized to GAPDH in IHH **(B)** and HepG2 **(E)** cells. **(C)** Fatty acid uptake in IHH hepatocytes. Results are expressed as Mean ± SEM. *P < 0.05, **P < 0.01 indicates a significant difference compared to control cells.

Hepatic steatosis is a key driver of NASH development. In order to determine whether cellular senescence affects hepatic lipid accumulation, lipid content was measured in senescent and control hepatocytes, in the absence or in the presence of oleic acid (OA) for 24h, in order to mimic pro-steatotic conditions. Hepatocytes treated with Nut, Eto and Doxo for 5 days displayed an increase in Oil Red O staining compared to control cells. In conditions with OA, the increased accumulation of lipids in senescent cells was even more pronounced (**Figures 2A, B, D, E**). Quantification of intracellular triglycerides (TG) confirmed the increase in lipid content in senescent cells (2,5-fold increase in Nut condition, 4,7-fold in Eto condition and 7,4-fold in Doxo condition in IHH, and 3,4-fold in Nut condition, 7-fold in Eto condition and 7,5-fold in Doxo condition in HepG2; **Figures 2C–F**). Accumulation of lipids in senescent hepatocytes was associated with increased mRNA and protein levels of several fatty acid transporters such as CD36, FATP2, FATP4, and FATP5 (**Figures 3A, B, D, E**). Similar results on lipid content and expression of fatty acid transporters were obtained when cells were treated with senescence-inducing drugs for 3 days (**Supplementary Figure 2**). Fatty acid uptake, quantified with radiolabeled oleate ^14^C, was increased 2,5 to 6 fold in senescent cells compared to control IHH cells (**Figure 3C**). Taken together, these results suggest that in human hepatocytes, cellular senescence increases expression of lipid transporters and increases lipid uptake capacity, resulting in hepatic steatosis.

### Cellular senescence increases β-oxidation and VLDL production, and decreases *de novo* lipogenesis in human hepatocytes

**Figure 4:**
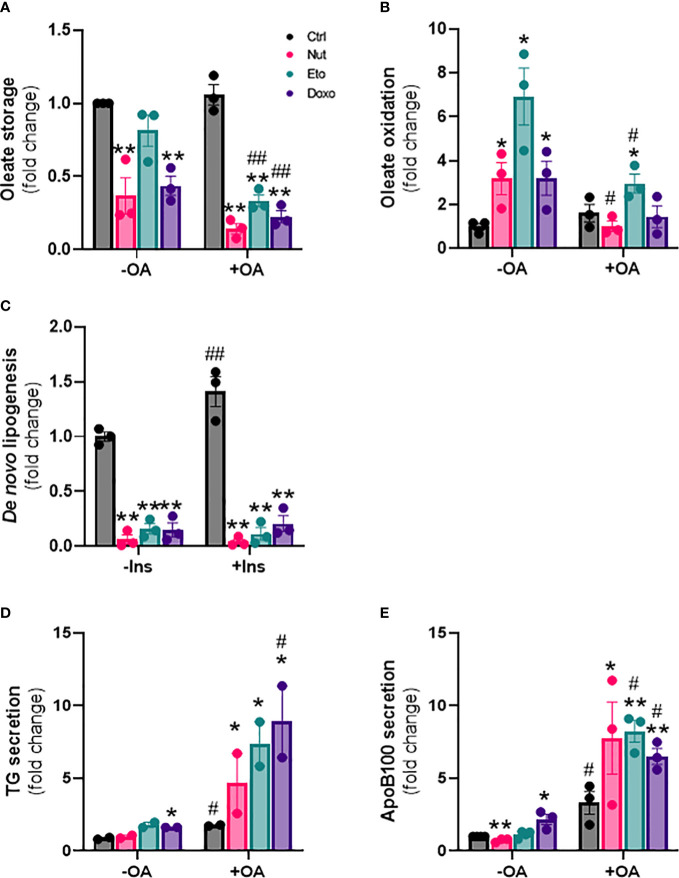
Cellular senescence decreases *de novo* lipogenesis and increases β-oxidation and VLDL production in human hepatocytes. IHH cells were treated with nutlin (Nut; 3 µM), etoposide (Eto; 20 µM) and doxorubicin (Doxo; 0,2 µM) for 5 days, with or without the addition of oleic acid (OA) for another 24h. **(A)** Oleate storage in IHH cells. Results were normalized to total protein levels. **(B)** β-oxidation in IHH cells. Results were normalized to total protein levels. **(C)** *De novo* lipogenesis in IHH cells with or without stimulation with insulin (100 nM) for 4h. **(D)** triglyceride and **(E)** apoB100 levels in IHH cell culture media. Results are expressed as Mean ± SEM. *P < 0.05, **P < 0.01 indicates a significant difference compared to control cells. ^#^P < 0.05, ^##^P < 0.01 indicates a significant difference compared to similar treatment condition without OA.

In addition to changes in lipid uptake, hepatic steatosis can also be due to changes in *de novo* lipogenesis (formation of lipids from glucose), fatty acid oxidation, and/or lipid secretion. To measure fatty acid storage, cells were incubated with radiolabeled oleate ^14^C for 4h, and oleate ^14^C incorporation into intracellular lipids was measured after extraction and separation of aqueous and lipid phases. Surprisingly, diversion of fatty acids towards lipid storage was decreased by 20-80% in senescent cells compared to control cells. This decrease in lipid storage capacity by senescence was exacerbated in the presence of OA (**Figure 4A**). Fatty acid oxidation capacity, assessed by measuring ^14^CO2 release after incubation with radiolabeled oleate, was increased 3 to 7 fold with senescence treatments without OA compared to control cells (**Figure 4B**). In the presence of OA, lipid oxidation was only increased ~2 fold in Eto-treated cells.

*De novo* lipogenesis was assessed by measuring incorporation of ^14^C glucose into intracellular lipids, in the presence or in the absence of insulin. As expected, *de novo* lipogenesis was increased in the presence of insulin in control cells. Surprisingly however, *de novo* lipogenesis was robustly decreased by 80-95% in senescent cells compared to control cells, both in the presence or in the absence of insulin (**Figure 4C**). Last, we also investigated the capacity of hepatocytes to produce lipids in the form of VLDL, by quantifying the amount of TG and of ApoB100, an apolipoprotein necessary for the assembly and secretion of VLDL, in the cell culture medium. We found increased levels of both TG and ApoB100 in cell culture medium from senescent cells compared to control cells, especially in the presence of oleic acid, indicating increased VLDL secretion (**Figures 4D, E**). Taken together, these results indicate that cellular senescence profoundly modifies glucose and lipid metabolism in hepatocytes, with increased lipid uptake capacity, but with fatty acids diverted towards oxidation rather than storage, decreased rates of *de novo* lipogenesis, and increased lipid secretion in the form of VLDL.

### Senescence increases glycogen content, glucose production and glucose oxidation in human hepatocytes

**Figure 5:**
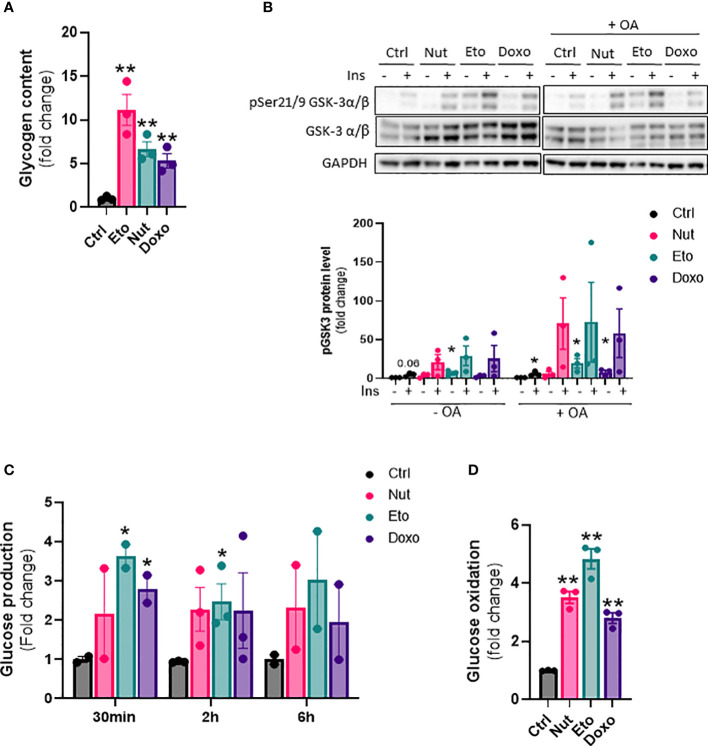
Increased glycogen content, glucose oxidation and hepatic glucose production in senescent human hepatocytes. IHH cells were treated with nutlin (Nut; 3 µM), etoposide (Eto; 20 µM) and doxorubicin (Doxo; 0,2 µM) for 5 days. **(A)** Glycogen content in IHH cells normalized to total protein levels. **(B)** Western blot analysis and quantification of pGSK3 (Ser21/9) normalized to total GSK3 levels in IHH cells treated with senescence-inducing drugs for 5 days, with or without the addition of oleic acid for another 24h, and acutely stimulated with insulin (10 nM). **(C)** Glucose levels in IHH cell culture medium after 30 min, 2h and 6h of incubation. **(D)** Glucose oxidation in IHH cells normalized to total protein content. Results are expressed as Mean ± SEM. *P < 0.05, **P < 0.01 indicates a significant difference compared to control cells.

Next, we wanted to investigate the effect of cellular senescence on glucose metabolism. Glycogen content was increased 5 to 11 fold in senescent IHH cells compared to control cells (**Figure 5A**). This was associated with an increase in glycogen synthase kinase 3 alpha and beta phosphorylation at Ser21 and Ser9, both in basal and insulin-stimulated conditions, and also with or without of oleic acid treatment (**Figure 5B**). To measure the hepatic glucose production capacity of senescent hepatocytes, we quantified glucose secretion in glucose-free culture medium over time. We found glucose levels to be increased by 2-3 fold in the medium from senescent compared to control cells (**Figure 5C**). The increase in glucose produced from senescent cells was observed at all-time points measured, but there was no increase in glucose levels over time, indicating a level of steady state and a balance between glucose production and utilization (**Figure 5C**). Lastly, we quantified glucose oxidation by measuring ^14^CO_2_ accumulation in the cell culture medium after treatment with radiolabeled ^14^C glucose. Glucose oxidation capacity was increased 3-5 fold in senescent cells compared to control cells (**Figure 5D**). Taken together, our results show that senescence causes changes in glucose metabolism in hepatocytes, including increased glycogen content, increased hepatic glucose output, as well as increased glucose oxidation capacity.

### Insulin and/or insulin resistance increases senescence but senescence increases insulin sensitivity in human hepatocytes

**Figure 6:**
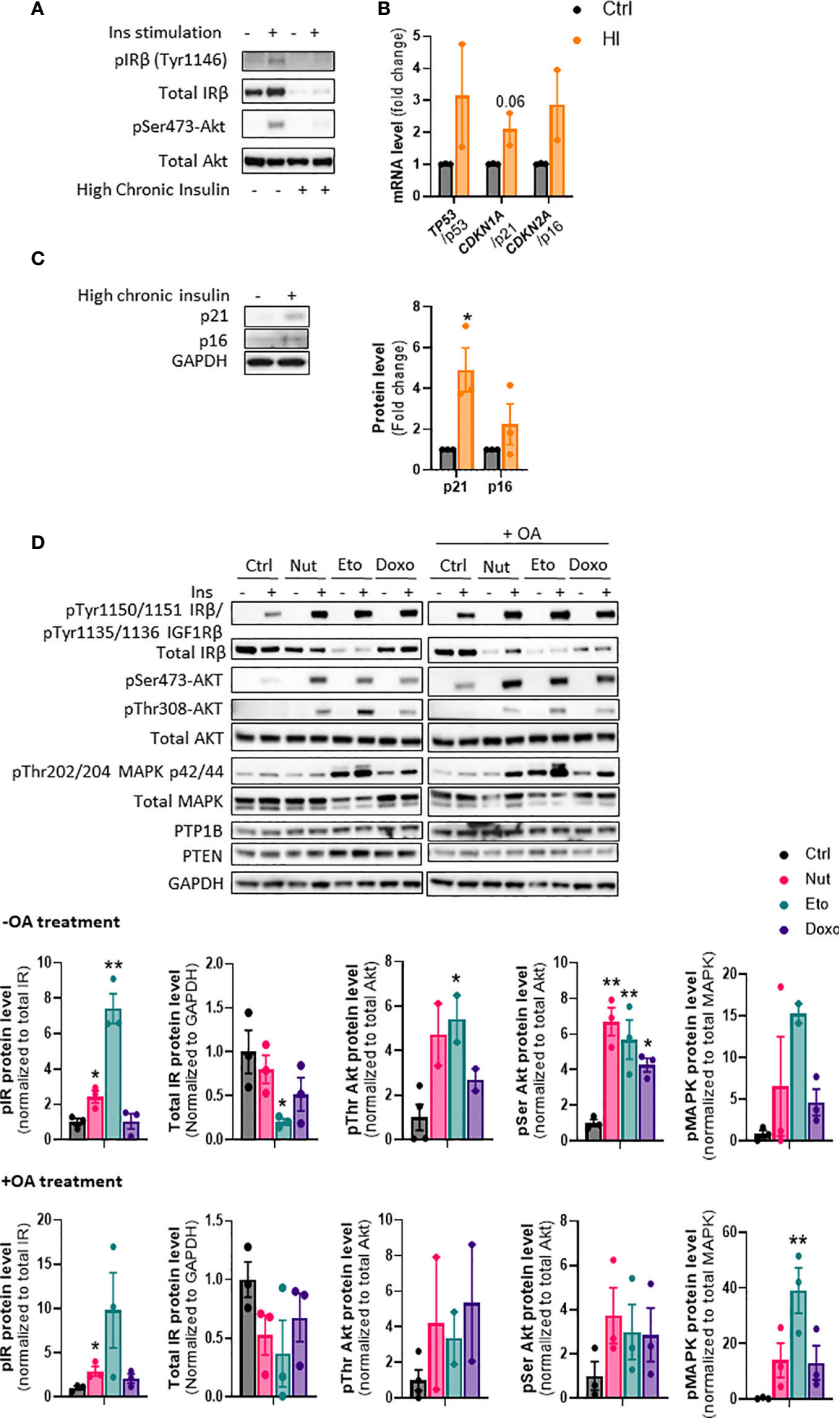
Cellular senescence increases insulin sensitivity in human hepatocytes. **(A)** IHH cells were chronically treated with high insulin levels (100 nM) for 5 days to induce insulin resistance. Western blot analysis of insulin receptor (IR) Tyr1146 and Akt Ser473 phosphorylation following acute 10 min insulin (10 nM) stimulation. **(B)** mRNA expression of senescence markers normalized to GAPDH after 5 days of culture with high insulin levels. **(C)** Western blot analysis and quantification of senescence markers p21 and p16 normalized to GAPDH, in IHH cells after 5 days of culture with high insulin levels. **(D)** IHH cells were treated with senescence-inducing drugs for 5 days, with or without the addition of oleic acid for another 24h, and acutely stimulated with insulin (10 nM) for 10 min. Western blot analysis and quantification of proteins involved in the insulin signaling pathway, in the insulin-stimulated conditions. GAPDH image is similar to that of **Figure 5B** as levels of different proteins were measured using the same membranes. Results are expressed as Mean ± SEM. *P < 0.05, **P < 0.01 indicates a significant difference compared to control cells.

Cellular senescence is associated with NAFLD and type 2 diabetes, which are also closely linked to insulin resistance (23). However, it is not known if insulin resistance could drive cellular senescence or if cellular senescence can contribute to insulin resistance. To induce insulin resistance *in vitro*, we treated IHH hepatocytes with high concentrations of insulin (24, 25). As expected, chronic high insulin treatment for 5 days led to a robust decrease in insulin receptor levels, and an almost complete inability to acutely respond to insulin, as measured by phosphorylation of Akt (**Figure 6A**). Chronic insulin treatment led to a robust increase in p21 protein levels, as well as an increase in p21, p53, and p16 mRNA levels (**Figures 6B, C**). Similar results on mRNA and protein levels of senescence markers were obtained when cells were treated chronically with high level of insulin for 3 days (**Supplementary Figures 3A, B**). This is in line with our recent results showing that chronic hyperinsulinemia promotes human hepatocyte senescence (26).

To determine if cellular senescence contributes to insulin resistance in hepatocytes, we treated IHH cells with the senescence-inducing drugs for 5 days, and measured the phosphorylation of key proteins of the insulin signaling pathway after acute insulin stimulation. As anticipated, acute insulin treatment led to a strong increase in phosphorylation of the insulin receptor (IR pTyr1150/1151 sites), Akt (Ser473 and Thr308 sites) and MAPK (Thr202 and 204 sites) proteins in control cells (**Figure 6D**). Surprisingly, cellular senescence did not cause insulin resistance, but rather robustly enhanced the insulin-stimulated phosphorylation of IR, Akt and MAPK, both in the absence and in the presence of oleic acid in the medium (**Figure 6D**). This occurred despite an overall decrease in IR protein levels. Similar increase in insulin signaling was obtained in HepG2 cells (**Supplementary Figure 4**). The increased insulin signaling observed was not due to a decrease in expression of PTP1B and PTEN, two important negative modulators of insulin signaling (**Figure 6D**). A similar increase in insulin sensitivity was observed when cells were treated with senescence-inducing drugs for 3 days (**Supplementary Figure 5**). Taken together, these results indicate that high insulin levels and/or the associated insulin resistance, can contribute to the development of cellular senescence. On the other hand, cellular senescence in hepatocytes leads to increased insulin sensitivity, suggesting that senescence acts as a brake on insulin resistance in hepatocytes, and that cellular senescence is rather a consequence than a cause of insulin resistance in hepatocytes.

## Discussion

Cellular senescence has recently been linked to several diseases, including metabolic diseases (27). To investigate whether senescence could contribute to NASH pathogenesis, we treated human hepatocytes with drugs known to induce cellular senescence such as nutlin-3a, etoposide or doxorubicin, which resulted in a robust increase in several senescence markers. Hepatic senescence was associated with an increase expression of fatty acid transporters, lipid uptake, and hepatic steatosis. Interestingly, senescent hepatocytes displayed enhanced insulin sensitivity compared to control hepatocytes. Lipid and glucose metabolism were also altered with senescence, with an increase in glycogen content, decrease in *de novo* lipogenesis, increase in VLDL-TG secretion from hepatocytes, and a paradoxical increase in glucose and fatty acid oxidation.

In this study, we used two different human hepatocyte cell lines, HepG2, a carcinoma cell line, and IHH, a hepatocyte cell line with closer characteristics to primary hepatocytes, especially related to fatty acid metabolism (15). We also used compounds that induce senescence through different mechanisms: nutlin-3a is an MDM2-antagonist and p53 activator, while etoposide and doxorubicin are both DNA damaging agents resulting in increased genotoxic stress for the cells (19, 20). We found consistent increases in senescence markers β-galactosidase, p53, p21 and p16 at both mRNA and protein level, and consistent phenotypic changes when treating both IHH and HepG2 cells with the different drugs for either 3 or 5 days. This increases the confidence in the physiological relevance of our findings, and in the association between the phenotypic changes and cellular senescence.

NAFL and NASH are closely associated with insulin resistance and T2D (28, 29). T2D is one of the strongest risk factors for a fast progression from NAFL to NASH (30) and NAFLD aggravates hepatic and peripheral insulin resistance which can promote the development of T2D (31). T2D is also associated with an increase in senescent cells in metabolic tissues such as liver and adipose tissue (32). We thus wondered whether cellular senescence could contribute to the development of insulin resistance in hepatocytes, or if insulin resistance contributes to the development of cellular senescence. Insulin resistance was induced by treating IHH cells with high levels of insulin. This caused a robust decrease in insulin receptor levels and downstream signaling, as reported previously (24, 33, 34). This was associated with an increase in senescence markers indicating that hyperinsulinemia and/or insulin resistance may contribute to the development of senescence in hepatocytes. This is in line with our recent finding that insulin drives senescence in human adipocytes (35). Surprisingly, we found that cellular senescence did not cause insulin resistance in hepatocytes, but rather increased insulin sensitivity, as measured by the robust increase in insulin receptor signaling following acute insulin stimulation (pIR, pAkt, pMAPK and PTP1B). This suggests that cellular senescence does not contribute to the insulin resistance associated with NASH or diabetes, but rather that senescence alleviates hepatic insulin resistance observed *in vivo*, which is thus caused by other mechanisms. This also suggests that senescence contributes to the worsening of hepatic steatosis, *via* both direct mechanisms, and *via* increased lipogenic action of insulin. Hepatocyte senescence could contribute indirectly to whole-body insulin resistance however, by favoring hepatic steatosis and VLDL secretion.

One of the most striking findings in our study was the robust increase in lipid content in senescent hepatocytes, which was further increased in pro-steatotic conditions in the presence of OA, and was due to an increase in lipid uptake. This suggests that cellular senescence contributes to NAFL and NASH development, as liver steatosis is one of the key features of NASH and an increase in cellular senescence has recently been associated with NASH (11, 36) (Baboota et al. Nature Metabolism, in press). Paradoxically, we observed a robust decrease in *de novo* lipogenesis capacity, increased fatty acid oxidation capacity and increased TG secretion in senescent hepatocytes, changes that would favor a decrease in hepatocyte lipid content. This suggests that quantitatively, lipid uptake far outweighs the changes in *de novo* lipogenesis and fatty acid oxidation, and is the major driver of senescence-induced hepatic steatosis. However, it is also possible that some changes in hepatocyte metabolism are compensatory changes occurring as a consequence of the increased steatosis. Indeed, we measured changes in metabolism after 5 days of compound treatment, and our results may not reflect what occurred in the early phases leading to that point. Most importantly, we are measuring capacity in specific assay conditions, which may be different from what is actually occurring over time, under the influence of different factors and hormones. Insulin in particular, present in the serum, may play a key role in the increased lipid accumulation over time, as it is a pro-lipogenic hormone and we observed increased insulin sensitivity in senescent hepatocytes compared to control hepatocytes. Indeed, most of our acute experiments have been performed in low serum conditions, and in the absence of exogenous insulin. Lastly, our study investigates the consequence of cellular senescence in human hepatocytes specifically, but does not take into account the potential contribution of other liver cell types such as kupffer cells, stellate cells and endothelial cells, which also play key roles in NASH development and progression. Nevertheless, our results recapitulate many changes that are seen in human NAFLD, obesity, or diabetes. Increased lipid uptake and increased expression of CD36 is observed in the liver in (2, 37, 38). Increased liver fatty acid oxidation has also been reported in NASH or obesity (39–41). Increased TG and VLDL secretion is increased in obese patients with NAFLD (42). Finally, despite the increased insulin sensitivity, we observed increased hepatic glucose production in senescent cells which is a key feature of diabetes and contributes to elevated glucose levels (43). However, as discussed above, hepatic glucose production was measured in the absence of exogenous insulin and in low serum conditions, and this may represent hepatic glucose output in “fasting” conditions in which insulin does not play a role. Hepatocyte ballooning is a key feature of human NASH. As senescent cells are characterized by enlarged and flattened cell morphology, it is tempting to speculate that ballooned hepatocytes may represent senescent hepatocytes with altered metabolism.

Taken together, our results show that cellular senescence in hepatocytes causes important changes in glucose and lipid metabolism, leading to increased steatosis, and increased glucose and TG production. Senescence may thus play a causal role in the development and progression of NAFLD, and targeting cellular senescence may represent a promising therapeutic approach for the treatment of NASH.

## Data availability statement

The original contributions presented in the study are included in the article/**Supplementary Material**. Further inquiries can be directed to the corresponding author.

## Author contributions

LB and JB designed experiments; LB, IA, RB, TK, JO, US and JB collected and/or analyzed and interpreted experimental data; LB, IA and JB wrote the paper; LB and IA prepared the figures; All authors critically reviewed and edited the manuscript; JB supervised the study. All authors contributed to the article and approved the submitted version.

## Acknowledgments

We acknowledge financial support from the Knut and Alice Wallenberg Foundation, the Swedish Foundation for Strategic Research, and the Wallenberg Centre for molecular and translational medicine, University of Gothenburg, Sweden. We thank Dr. Silvia Gogg, Dr. Birgit Gustafson and Annika Nerstedt from the Lundberg Laboratory for technical help. We thank Dr. Mathieu Cinato from Wallenberg laboratory for technical help.

## Conflict of interest

Authors TK, IA, JO and JB are or were shareholders and employed by Astrazeneca. JB is currently employed by and a shareholder of Evotec.

The remaining authors declare that the research was conducted in the absence of any commercial or financial relationships that could be construed as a potential conflict of interest.

## Publisher’s note

All claims expressed in this article are solely those of the authors and do not necessarily represent those of their affiliated organizations, or those of the publisher, the editors and the reviewers. Any product that may be evaluated in this article, or claim that may be made by its manufacturer, is not guaranteed or endorsed by the publisher.

## Supplementary material

Supplementary Table 1 Primer sequences.

Supplementary Table 2 List of antibodies.
