# Supplementary material for: Cellular senescence in hepatocytes contributes to metabolic disturbances in NASH
Source: Front Endocrinol (Lausanne). 2022 Aug 22;13:957616. doi: 10.3389/fendo.2022.957616 (PMC9441597; doi:10.3389/fendo.2022.957616)
Supplement: Supplementary Table 1 — Primer sequences. [file Table_1.pdf]

Supplemental Table 1

Primer sequences

Gene	Species	Forward sequence	Reverse sequence
**CD36**	Human	TTGATGTGCAAAATCCACAGG	TGTGTTGTCCTCAGCGTCCT
**GAPDH**		ACAACTTTGGTATCGTGGAAGG	GCCATCACGCCACAGTTTC
**GLB1**		TATACTGGCTGGCTAGATCACTG	GGCAAAATTGGTCCCACCTATAA
**CDKN1A**		TGTCCGTCAGAACCCATGC	AAAGTCGAAGTTCCATCGCTC
**SERPINE 1**		GCACCACAGACGCGATCTT	ACCTCTGAAAAGTCCACTTGC
**SLC27A2**		TGGTGTCGCCAGAACTACAAG	GAAAGAGTCAATCCCATCTGTGT
**SLC27A5**		TGATGGGACTTGTCGTTGG	CCAGAAGCAGGAAGTAGAGAAC
**SLC27A4**		CGGTTCTGGGACGATTGTAT	AACCTGGTGCTGGTTTTCTG
**TP53**		CCCCTCTGAGTCAGGAAACAT	GCATTCTGGGAGCTTCATCTG
