# Supplementary material for: Cellular senescence in hepatocytes contributes to metabolic disturbances in NASH
Source: Front Endocrinol (Lausanne). 2022 Aug 22;13:957616. doi: 10.3389/fendo.2022.957616 (PMC9441597; doi:10.3389/fendo.2022.957616)
Supplement: Supplementary Table 2 — List of antibodies. [file Table_2.pdf]

Supplemental Table 2

List of antibodies

**Antibody**	**Source**	**Cat. no.**
Akt	Cell signaling	#9272
Phospho Akt Ser473	Cell signaling	#9271
Phospho Akt Thr308	Cell signaling	#9275S
CD36	Cell signaling	#14347
FABP1	Proteintech	13626-1-AP
FATP2	Abcam	ab83763
FATP4	Proteintech	11013-1-AP
FATP5	Sigma-Merck	HPA007292
GAPDH	Santa cruz	sc-47724
GSK-3 a/b (D75D3)	Cell signaling	#5676T
Phospho GSK-3α/β (Ser21/9)	Cell signaling	#9331
IRβ	Santa cruz	sc:711
Phospho IGF1 Recpβ (Tyr1131)/IRβ (Tyr1146)	Cell signaling	#3021
Phospho IGF-I Receptor β (Tyr1135/1136)/Insulin Receptor β (Tyr1150/1151)	Cell signaling	#3024
MAPK Erk1/2	Millipore	#06/182
Phospho p44/42 MAPK Erk1/2	Cell signaling	#4370
Anti-mouse IgG	Cell signaling	#7076
PAI-1	Abcam	ab66705
PTEN	Cell signaling	#07-1016
PTP1B	Oncongene	PH01-100uG
p16-INK4A	Proteintech	10883-1-AP
p21 Waf1/Cip1	Santa cruz	sc-6246
p53	Cell signaling	#2425S
Anti-rabbit IgG	Cell signaling	#7074
