# Supplementary figures and images for: Cellular senescence in hepatocytes contributes to metabolic disturbances in NASH
Source: Front Endocrinol (Lausanne). 2022 Aug 22;13:957616. doi: 10.3389/fendo.2022.957616 (PMC9441597; doi:10.3389/fendo.2022.957616)

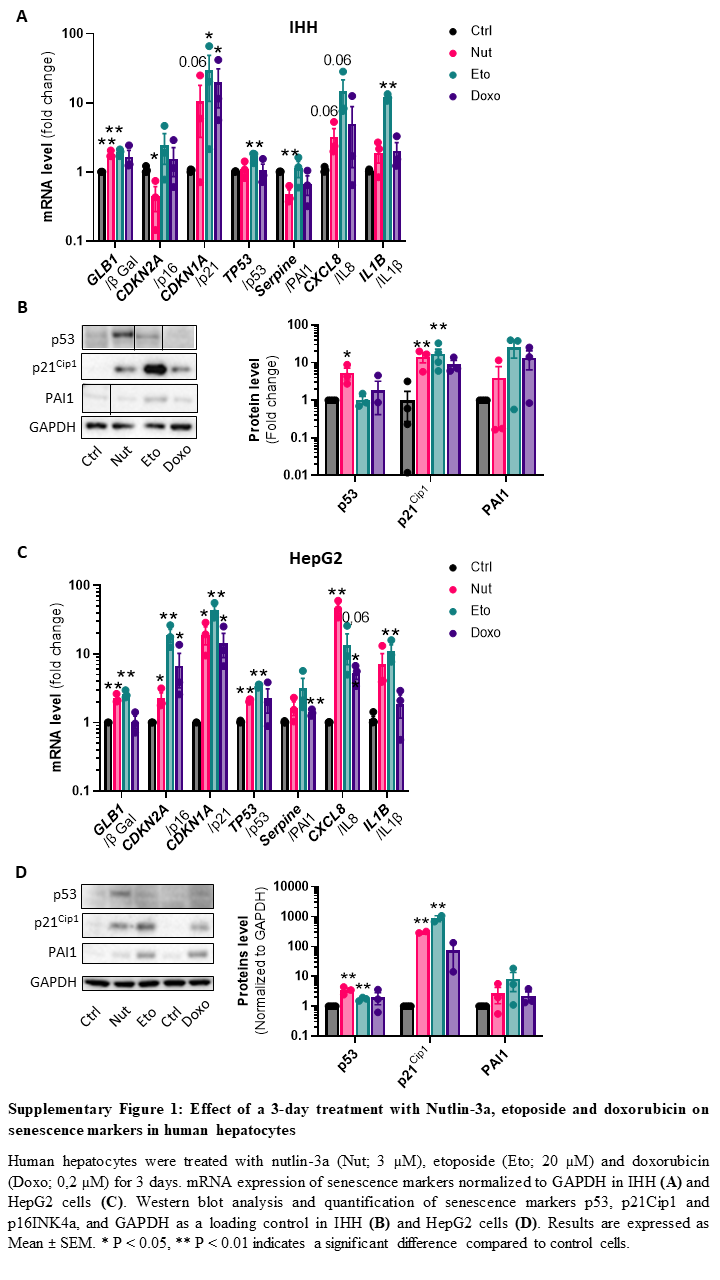

Supplement: Supplementary file 3 [file Image_1.tif]

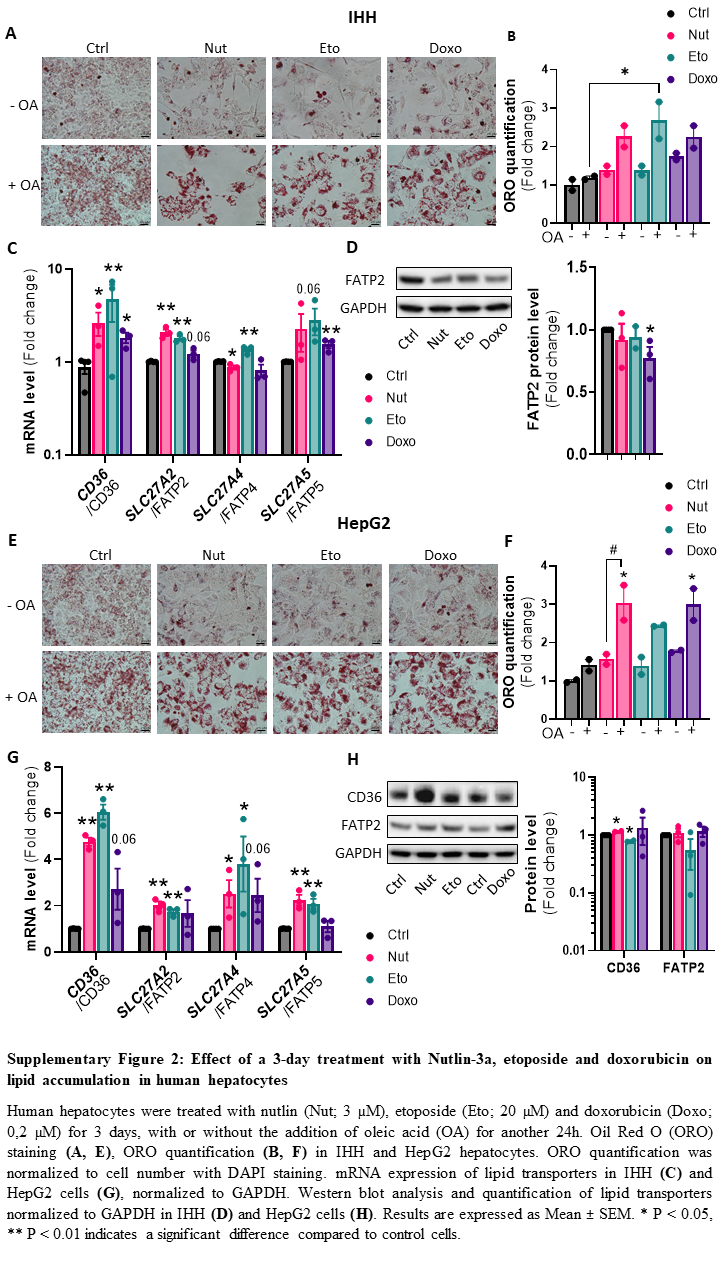

Supplement: Supplementary file 4 [file Image_2.tif]

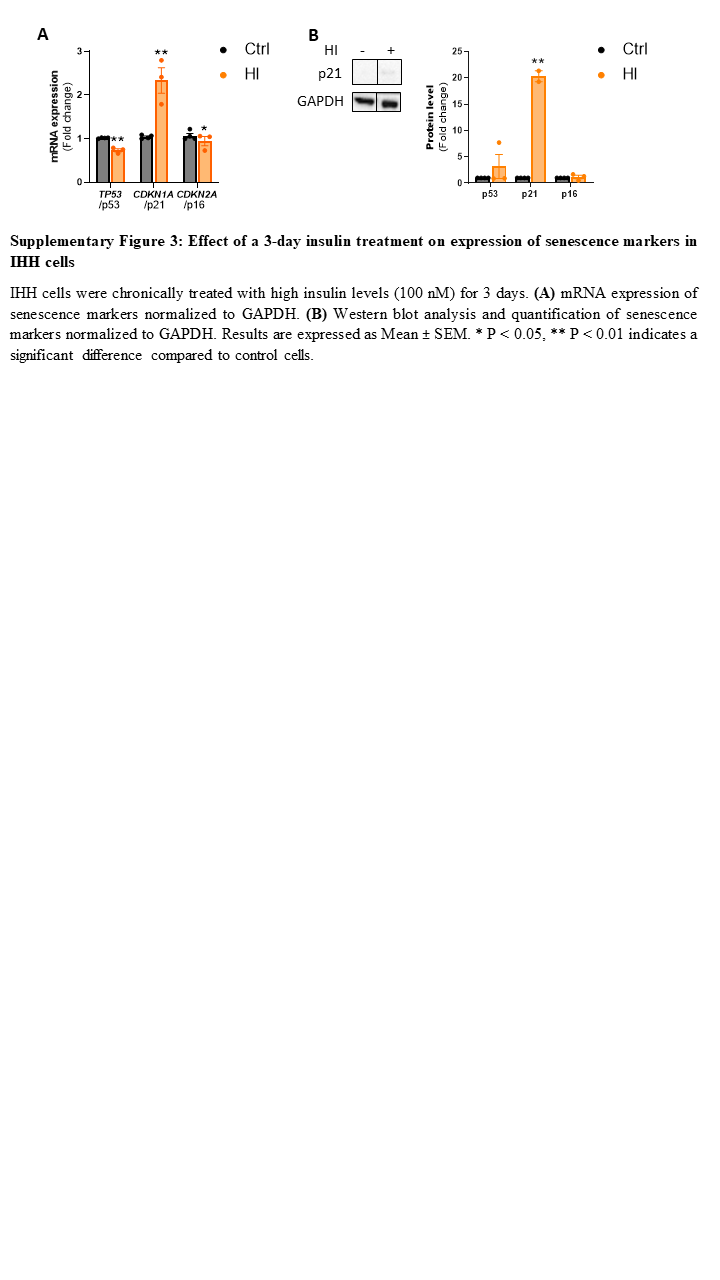

Supplement: Supplementary file 5 [file Image_3.tif]

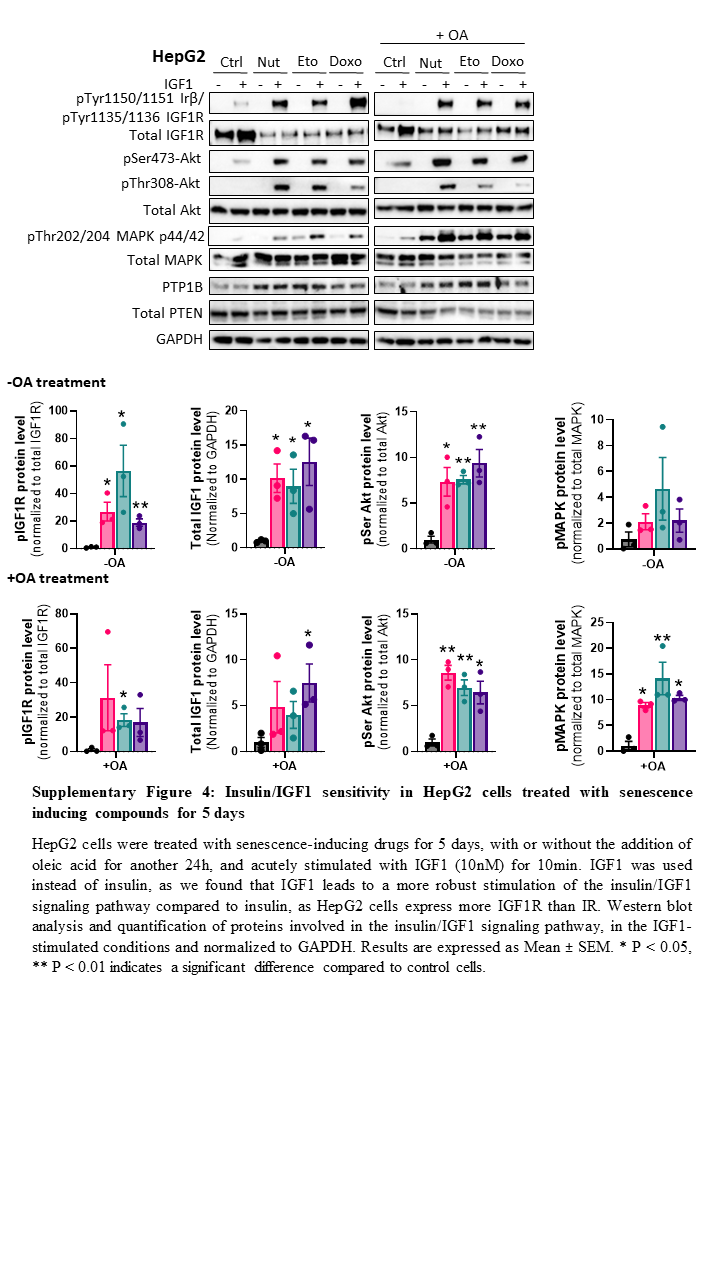

Supplement: Supplementary file 6 [file Image_4.tif]

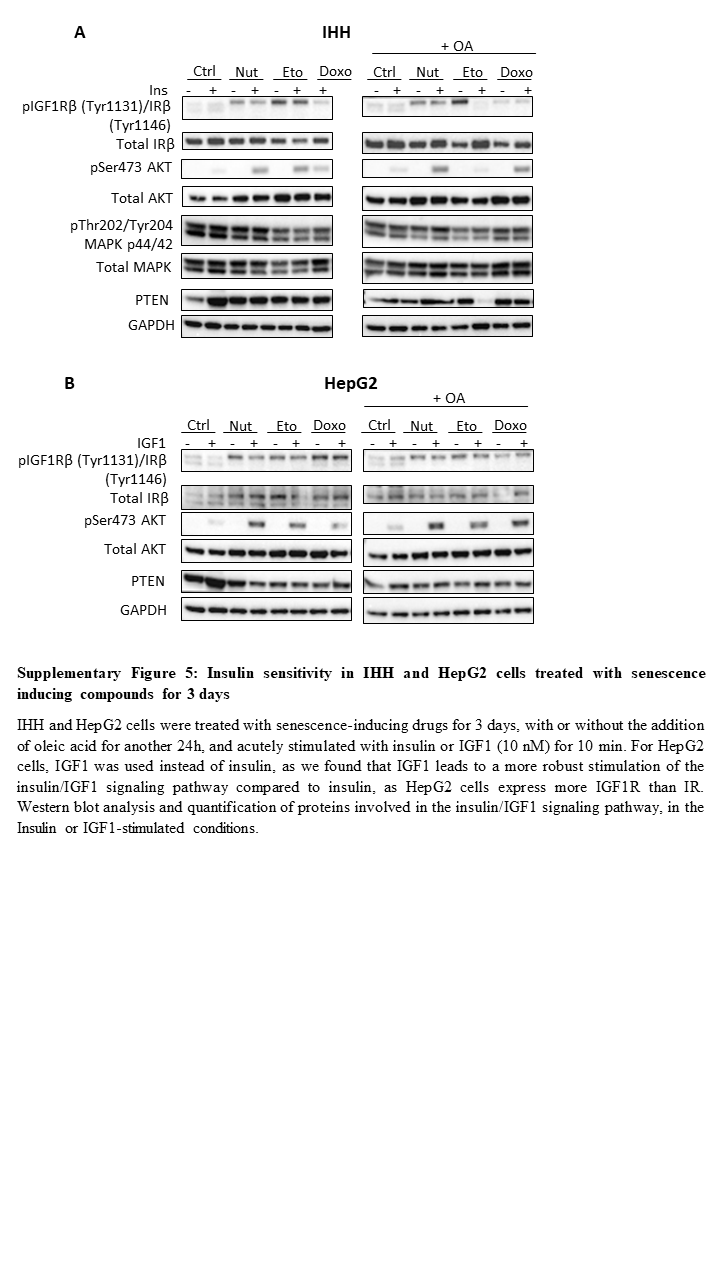

Supplement: Supplementary file 7 [file Image_5.tif]
